# Potential business model for a European vaccine R&D infrastructure and its estimated socio-economic impact

**DOI:** 10.12688/f1000research.141399.1

**Published:** 2023-10-24

**Authors:** Stefan Jungbluth, William Martin, Monika Slezak, Hilde Depraetere, Carlos A. Guzman, Anton Ussi, David Morrow, Fran Van Heuverswyn, Sven Arnouts, Manuel J. T. Carrondo, Ole Olesen, Tom H.M. Ottenhoff, H. M. Dockrell, Mei Mei Ho, Alexandre Dobly, Dennis Christensen, Joaquim Segalés, Fabrice Laurent, Frédéric Lantier, Norbert Stockhofe-Zurwieden, Francesca Morelli, Jan A.M. Langermans, Frank A.W. Verreck, Roger Le Grand, Arjen Sloots, Donata Medaglini, Maria Lawrenz, Nicolas Collin

**Affiliations:** 1 European Vaccine Initiative (EVI), Heidelberg, 69115, Germany; 2 Department of Vaccinology and Applied Microbiology, Helmholtz Centre for Infection Research (HZI), Braunschweig, 38124, Germany; 3 EATRIS- European Research Infrastructure for Translational Medicine, Amsterdam, 1081 HZ, The Netherlands; 4 Flanders Vaccine, Diepenbeek, 3590, Belgium; 5 provaxs - Ghent University, Merelbeke, 9820, Belgium; 6 iBET - Instituto de Biologia Experimental e Tecnológica, Oeiras, 2781-901, Portugal; 7 Department of Infectious Diseases, Leiden University Medical Center, Leiden, 2300RC, The Netherlands; 8 London School of Hygiene & Tropical Medicine (LSHTM), London, WC1E 7HT, UK; 9 Medicines and Healthcare products Regulatory Agency (MHRA), Potters Bar, Hertfordshire, EN6 3QG, UK; 10 Sciensano, Brussels, 1050, Belgium; 11 Statens Serum Institut (SSI), Copenhagen, 2300, Denmark; 12 Centre de Recerca en Sanitat Animal (CReSA, IRTA-UAB), Bellaterra, 08193, Spain; 13 Université François Rabelais de Tours, Centre Val de Loire, UMR1282 ISP, INRAE, Nouzilly, 37380, France; 14 Wageningen Bioveterinary Research, Wageningen University & Research (SWR), Wageningen, 6700 HB, The Netherlands; 15 CIRMMP, Sesto Fiorentino, Florence, 50019, Italy; 16 Biomedical Primate Research Centre (BPRC), Rijswijk, 2288 GJ, The Netherlands; 17 IDMIT Infrastructure, CEA, Université Paris-Saclay, Inserm, Fontenay-aux-Roses, 92265, Cedex, France; 18 Intravacc, Bilthoven, 3721 MA, The Netherlands; 19 University of Siena, Siena, 53100, Italy; 20 Vaccine Formulation Institute (VFI), Plan-les-Ouates, Geneva, 1228, Switzerland

**Keywords:** Vaccines, research and development, research infrastructure, business model, sustainability, impact assessment, science policy

## Abstract

**Background:**

Research infrastructures are facilities or resources that have proven fundamental for supporting scientific research and innovation. However, they are also known to be very expensive in their establishment, operation and maintenance. As by far the biggest share of these costs is always borne by public funders, there is a strong interest and indeed a necessity to develop alternative business models for such infrastructures that allow them to function in a more sustainable manner that is less dependent on public financing.

**Methods:**

In this article, we describe a feasibility study we have undertaken to develop a potentially sustainable business model for a vaccine research and development (R&D) infrastructure. The model we have developed integrates two different types of business models that would provide the infrastructure with two different types of revenue streams which would facilitate its establishment and would be a measure of risk reduction. For the business model we are proposing, we have undertaken an ex ante impact assessment that estimates the expected impact for a vaccine R&D infrastructure based on the proposed models along three different dimensions: health, society and economy.

**Results:**

Our impact assessment demonstrates that such a vaccine R&D infrastructure could achieve a very significant socio-economic impact, and so its establishment is therefore considered worthwhile pursuing.

**Conclusions:**

The business model we have developed, the impact assessment and the overall process we have followed might also be of interest to other research infrastructure initiatives in the biomedical field.

## Introduction

Vaccines are amongst the most effective public health tools available to humanity to fight infectious diseases ( Greenwood, 2014; Doherty *et al.*, 2016). Outstanding achievements of vaccines include the control or eradication of several previously devastating human and veterinary diseases such as smallpox and rinderpest (both eradicated), the near eradication of poliomielitis, and, importantly, a significant reduction of the global negative consequences of the COVID-19 pandemic on global health, society and economy. Despite these successes, there are numerous gaps and weaknesses in our current vaccine arsenal, and considerable work and innovation is needed to reduce the threat and burden of endemic and emerging infectious diseases, as well as to prepare for unknown future threats. However, vaccine development is a time-consuming and complex process that involves progression through various phases, from discovery and preclinical research to clinical development and large scale manufacturing of the final vaccines. All these steps require significant financial resources and broad technical capabilities. Development of vaccines is also very unpredictable as vaccine candidates can fail during any of the above-mentioned, increasingly expensive stages. On average, less than 1 out of 10 vaccine candidates in preclinical development eventually reach the market.

In a previous analysis based on structured feedback from experts, we identified numerous deficiencies in the European vaccine R&D landscape ( Jungbluth *et al.*, 2022). Many of these needs could be addressed by a well-designed, public health-driven and sustainable vaccine R&D infrastructure that—by the provision of services, expertise, facilities and other types of support—could foster innovation and scientific advancements in the larger vaccine R&D space ( Leroy *et al.*, 2014; IPROVE, 2016).

Research infrastructures (RIs) are physical and organizational structures, facilities, resources, and services that support scientific research and innovation. These infrastructures are designed to provide scientists, researchers, and innovators with the necessary resources, collaborative environments and expertise to conduct cutting-edge research and address complex scientific questions. RIs can vary widely in scope, scale, and focus, but are typically long-term initiatives with lifespans of decades rather than years ( OECD, 2019). As the establishment and maintenance of RIs usually involve significant investments from governments, international organizations and private entities, the decision of whether to establish an RI is typically informed by a technical and conceptual feasibility study in which different RI design options are developed and compared, the expected socio-economic impacts (SEIs) assessed, and—eventually—detailed business and implementation plans prepared. In the present article, we describe the outcome of such a design study and SEI assessment for a future European vaccine R&D infrastructure. The aim of the feasibility study is to inform funders, policy and decision makers and other stakeholders about critical infrastructure needs within the vaccine R&D community and to provide a sound basis for evidence-based decision making regarding the establishment of a sustainable vaccine R&D infrastructure.

The feasibility study, including the findings described in the present article, were produced in the context of TRANSVAC (European Network of Vaccine Development and Research). TRANSVAC—an initiative that comprises three vaccine infrastructure projects funded by the European Union (EU) from October 2009 until April 2023—is a distributed, network-based research infrastructure integrating the expertise and facilities of 26 leading research organisations from ten European countries ^1^. Provided with a total cumulative funding of approximately 27 million EUR and a focus on prophylactic and therapeutic vaccines for human and veterinary use, TRANSVAC has supported vaccine R&D by developing and optimising scientific-technical services, which were then offered to the vaccine development community at no cost along with cutting-edge training in vaccinology.

## Methods

### Development of business model

Four different business model options were developed using the procedure described below, one of the models was a combination of two individual models developed. In the present article, we only describe the details and expected impacts for the business model option that was prioritized for further development (“hybrid model”) following the assessment of all models using the evaluation framework developed (see below). The working steps in the overall process were the following ones:

1. Defining the scope for business model design options:
  To determine what business model options should be considered in the assessment, an evaluation of market needs and of TRANSVAC’s current capabilities was conducted to identify key opportunity areas for a future sustainable vaccine RI. In addition to intelligence produced directly by TRANSVAC ( Leroy *et al.*, 2014; Jungbluth *et al.*, 2022; and unpublished), this step included the consultation of pertinent business ( IPROVE, 2016) and market intelligence ^2^ ^,^ ^3^, the analysis of different business models used by other existing Ris in the biomedical area ( ESFRI, 2021, and references and links therein), and surveys and expert interviews we conducted. The individuals that participated in this process included representatives from all major stakeholder groups considered relevant for this venture, including potential users of a future vaccine RI, funders and finance providers, policy- and decision makers, industry, international organizations, vaccine development alliances, as well as existing Ris in the biomedical area in Europe.
2. Creating an evaluation framework for assessment:
  A list of specific metrics for the quantitative scoring of the business models was developed. The metrics reflect important criteria, including unique selling position; competitive environment; financial upside; sustainability; risks, investment needs; complexity of implementation; capabilities/assets, and others.
3. Describing details of the design options:
  Based on the market gaps, needs and opportunities identified in step 1 (current vaccine market situation), and on the analysis of currently existing/used business models in a range of industries and several successful Ris, different business model archetypes were developed and put forward for further consideration in the next step.
4. Model evaluation and selection:

Evaluation and selection of the different business model options were carried out using the evaluation framework described.

The entire process as outlined above, including the overall suggested positioning of the sustainable vaccine infrastructure, was performed keeping in mind the gaps and needs previously identified as part of the TRANSVAC project, as well as the objectives, scope and missions of other (European) Ris already existing.

### Definition of impact dimensions

To select impact categories for analysis, a literature review on the socio-economic impact assessment practices was made first, followed by a benchmarking of different practices and information ( Reid *et al.*, 2015; ESFRI, 2019). Multiple key impact indicators emerged, with three main impact categories aligned with the literature and taking into consideration the following standard metrics:

- Health impact: Innovation for vaccine R&D, models developed, tools/technologies/solutions developed, vaccines developed and related data generation (immunological signatures/correlates of protection/efficacy and safety …)
- Societal impact: Dissemination activities and new publications, training and education, human resources
- Economic impact: Investments to accelerate vaccine development, impact on small- and medium sized enterprises (SMEs), revenues generated.

Applied to the vaccine research infrastructure case, these three dimensions draw a picture of how such an infrastructure would deliver socio-economic impacts, locally, regionally, and globally, and help to better understand how it could fulfil its long-term objectives. Moreover, these categories have in common that they capture general issues having a fundamental impact on a vaccine infrastructure, represent a systematic approach for quantification, and are widely applicable.

The three main dimensions of socio-economic impact that were analysed reflect the impact of vaccines which is broad and far-reaching, though not consistently quantifiable, analysed or communicated. Traditionally, the perceived benefits of vaccinations are to reduce morbidity and mortality from infections, and those remain the drivers for the innovation of new vaccines. However, an increasing appreciation for the economic and societal impact of vaccines is being included in the development and assessment of vaccine programmes, as they potentially deliver greater benefits to society ( Rodrigues and Plotkin, 2020; Beck *et al.*, 2022). In the assessment we have undertaken, the impacts across the three different dimensions have been estimated for the first 10 years of the RI’s period of operation.

### Key performance indicators (KPIs)

A series of KPIs was developed that was subsequently used for assessing the *ex ante* impact of the vaccine RI along the different dimensions described above ( Table 1). Eventually, once the vaccine RI is established and operational, the same KPIs could be used for the actual performance monitoring of the venture, as well as for a potential *ex post* impact assessment. For the actual performance monitoring of the vaccine RI once it is up and running, each KPI would need to be assessed with a specific frequency (quarterly or annually) and using a specific way of data collection (e.g. via operational and economic performance indicators, surveys, interviews, or from other external sources).

**Table 1.** Summary of proposed KPIs including their assessment frequency and time horizon.

No.	Impact dimension	Measure of potential impact	Assessment frequency	Time horizon of impact measurement
1a	Health	Expected number of new vaccines in clinical development	Annual	Long term (impact measures relate to five or more years from now)
1b	Health	Expected number of future deaths and severe cases prevented	Annual	Long term (impact measures relate to years 11 onwards)
1c	Health	Expected disability-adjusted life years (DALY) improvement	Annual	Long term (impact measures relate to years 11 onwards)
2a	Societal	Number of new jobs created (within subsidiary companies)	Quarterly	Long term (impact measures relate to five or more years from now)
2b	Societal	Percentage of researchers who have been through training who report an improvement in their knowledge base/knowledge capital created	Annual	Near term (annual impact)
2c	Societal	Media appearances (Television, radio, press, online)	Annual	Near term (annual impact)
2d	Societal	Number of research partners trained through vaccine infrastructure	Annual	Near term (annual impact)
3a	Economic	Expected revenue generated	Annual	Near-term and cumulative impact over ten years of operation
3b	Economic	Expected value of funding attracted, including services/grants	Annual	Near-term and cumulative impact over ten years of operation
3c	Economic	Expected value of licensing deals	Annual	Near-term and cumulative impact over ten years of operation
3d	Economic	Expected cash inflows at bio-holding and venture level	Quarterly	Near-term and cumulative impact over ten years of operation
3e	Economic	Number of SMEs created (this is also a proxy for number of licensing deals)	Annual	Long term (impact measures relate to five or more years from now)
3f	Economic	Number of scientific services provided	Annual	Near-term and cumulative impact over ten years of operation
3g	Economic	Number of new patents issued	Annual	Near-term and cumulative impact over ten years of operation
4a	Operational	Expected number of new publications	Annual	Near term (annual impact)
4b	Operational	Expected number of new scientific services established as an offering to researchers	Annual	Near term (annual impact)
4c	Operational	Number of vaccine projects supported	Annual	Near-term (annual impact) and cumulative impact over ten years of operation
4d	Operational	Organisation of stakeholder and investor meetings	Annual	Near term (annual impact)

For performance monitoring, three categories of key performance indicators (i.e., short-term impact, long-term cumulative impact, and operational metrics) were selected to mirror the progress of the RI in achieving established objectives and goals. With operational metrics, the functioning of infrastructure can be monitored, whereas the short-term impact and long-term cumulative impact indicators enable actual measurement of achievements over time and their communication to key stakeholders. Performance measures include the tracking of economic, societal and other health indicators of impact. These dimensions were chosen as they are also relevant for the mission we propose for a vaccine RI to accelerate vaccine development in the interest of public health and societal benefits.

Initially a larger list of approximately 30 KPIs was drawn up that subsequently was down-selected based on their relevance, credibility, quality as indicators and direct linkage to proposed vaccine R&D RI’s objectives. The KPIs have two major functions: firstly, they should quantify generated impact on a higher level to enable addressing a broader set of audiences and secondly, they should enable management of the specific areas on a more granular level.

### Impact assessment: Data sources and assumptions

Table 2 summarises the data sources and assumptions that were used for the estimation linked to the KPIs selected. For a complete list containing all KPIs please see Table 1.

**Table 2.** Sources and assumptions for different KPIs developed.

KPI	Assumptions and data sources used
**Estimated health impact**	
Expected number of new vaccines in clinical development	For the estimation, the following average numbers for duration and success rate at different development stages were used: **Table:** Preclinical Phase I Phase II References Duration input (years) 2 2 2 Terry *et al.* (2018); EVI-internal data (unpublished) Success rate input (%) 53 57 38 Terry *et al.* (2018); EVI-internal data (unpublished)
Expected number of future deaths and severe cases prevented	For high impact scenarios: data from COVID-19 vaccines were used for modelling ( Watson *et al.*, 2022; Wyper *et al.*, 2022) For low impact scenarios: data from influenza vaccines were used as a starting point for modelling ( Sah *et al.*, 2018)
Expected disability-adjusted life years (DALY) improvement	For high impact scenarios: data from COVID-19 vaccines were used for modelling ( Watson *et al.*, 2022; Wyper *et al.*, 2022) For low impact scenarios: data from influenza vaccines were used as a starting point for modelling ( Sah *et al.*, 2018)
**Estimated societal impact**	
Number of new jobs created (within subsidiary companies)	Based on an average number calculated from data sampling from online search at several biotech companies
Percentage of researchers who have been through training who report an improvement in their knowledge base/knowledge capital created	Based on numbers from TRANSVAC (unpublished)
Media appearances (Television, radio, press and online)	Based on numbers from TRANSVAC (unpublished)
Number of research partners trained through vaccine infrastructure	Based on numbers from TRANSVAC (unpublished)
**Estimated economic impact**	
Expected revenue generated Expected value of funding attracted, including services/grants Expected value of licensing deals	For the development costs and the exit values of vaccines, the following average numbers were used: **Table:** Preclinical Phase I Phase II References Average cost (€ million) 10 7 14 Terry *et al.* (2018); EVI-internal data (unpublished) Exit value (€ million) n.a. 77 211 Pharma Deals ^4^ Other revenue-related assumptions include: Total investment received by RI: €100 million Exit revenue for RI (% of deal value): 20% Management fee earned (% of invested money): 2% Brokerage fee for RI (average/year for 10 years): €160k Grant income (average/year for 10 years): €200k General and administrative costs (average/year for 10 years): 1.1€ million
Number of SMEs created (this is also a proxy for number of licensing deals)	A total number of 15 vaccine candidates are estimated to be in-licensed by the RI: 11 at preclinical stages, 3 at phase I, 1 at phase II
Number of scientific services provided	Based on numbers from TRANSVAC (unpublished)
Number of new patents issued	Based on numbers from TRANSVAC (unpublished)
**Estimated operational impact**	
Expected number of new publications	Based on numbers from TRANSVAC (unpublished)
Expected number of new scientific services established as an offering to researchers	Based on numbers from TRANSVAC (unpublished)
Number of vaccine projects supported	Based on numbers from TRANSVAC (unpublished)
Organisation of stakeholder and investor meetings	Based on numbers from TRANSVAC (unpublished)

## Results

### (i) Proposed business model for a European vaccine R&D infrastructure

The gaps-and-needs assessment of vaccine R&D in Europe we conducted previously had identified several opportunities for the conceptual design of a sustainable vaccine R&D infrastructure. These include general gaps and needs in the vaccine R&D field such as lack of expertise in transitioning vaccine candidates from preclinical research to clinical testing, as well as specific aspects including the lack of access to value chain services, such as vaccine formulation expertise, pre-clinical testing, small-scale manufacturing according to good manufacturing practices (GMP), regulatory support. Moreover, our analysis revealed the difficulty of securing funding or financing for late-stage preclinical vaccine development, vaccine GMP manufacturing and early clinical testing (for details, see Jungbluth *et al.*, 2022). Keeping in mind these and other findings from the gaps-and-needs analysis, according to our vision a future sustainable vaccine infrastructure should ideally address the following aspects:

- Provide access to a powerful network of researchers with vaccine development expertise and facilities that is able to assess potential risks related to the development of specific vaccines and that is able to offer support that will increase the probability of success of specific vaccine development projects
- Provide support/services, resources and capabilities for vaccine development
- Support horizontal themes across vaccine development (i.e. disease/pathogen-independent)
- Provide transversal and multi-disciplinary expertise in vaccine development and recommend experts in necessary fields
- Offer (seed) funding for vaccine research and development (R&D)
- Accelerate vaccine development in the interest of public health and societal benefits.

Based on these expectations defined, amongst the different business model options we developed, the one we selected for further consideration is described below.

- **Novel concept of European Infrastructure business model**

The option we are proposing is a combination of two different business models -namely a contract development partnership model and a bio-holding model- that are outlined below. This hybrid model was selected as it has the advantage of offering two different and independent types of revenue streams. Moreover, regarding the establishment of the RI, this hybrid model would facilitate the initial setting up of the infrastructure by starting with one of the models first (the contract development partnership) and subsequently implement the establishment of the other (the bio-holding model), which is the more challenging model to build.

- **Contract development partnership model**

**Figure 1.**
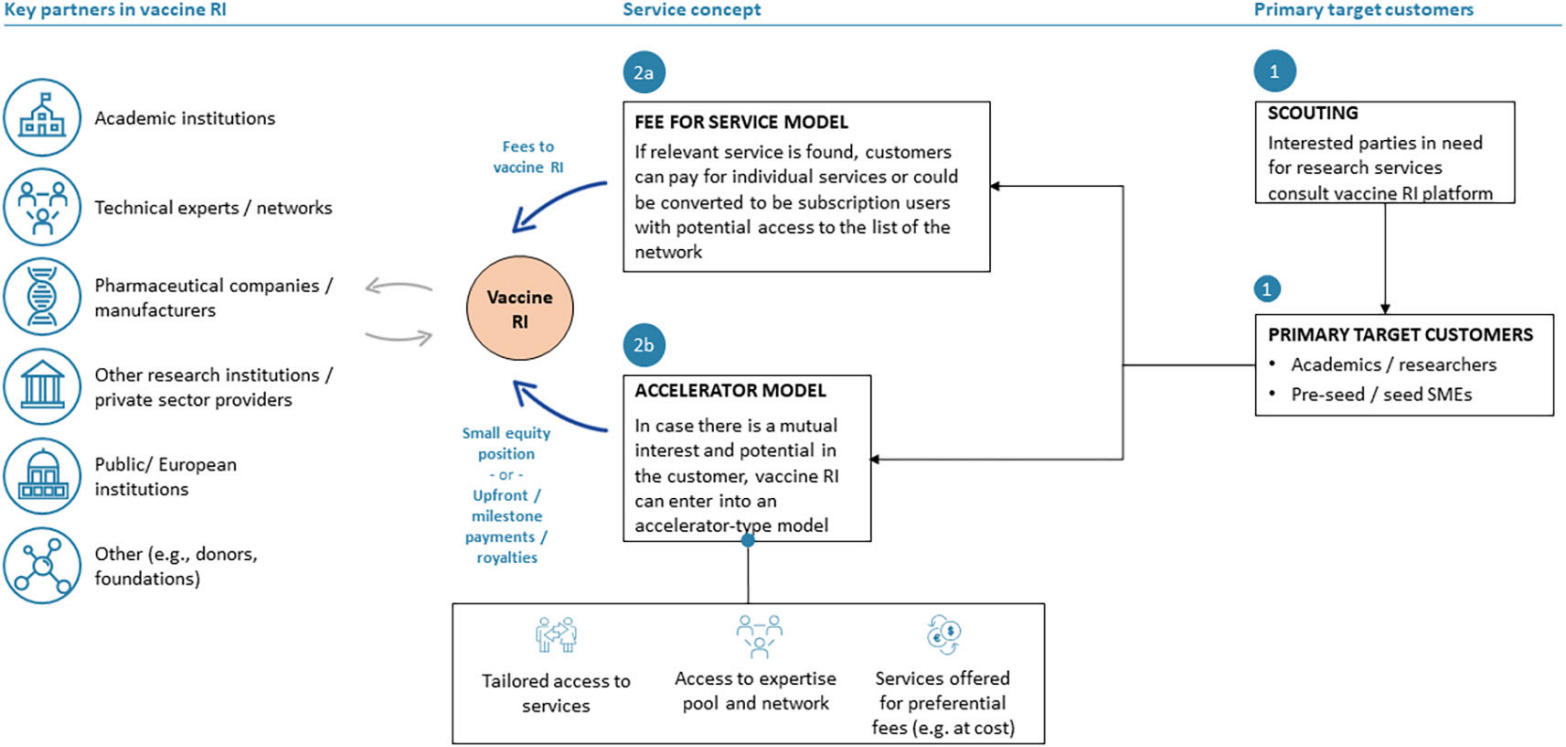
Overview of the functioning of the contract development partnership model within the vaccine RI based on the hybrid business model. Blue arrows indicate financial flows, grey arrows scientific-technical or other types of participation and contribution.

Under this business model option ( Figure 1), the RI will offer to its customers a full suite of tailored value-chain and transversal scientific-technical and other services related to vaccine development and business building. This model assumes no access to (cash) funding. However, funding may potentially be provided to customers through in-kind contributions by the RI providing free or discounted services to customers in exchange for a small stake in the customers’ vaccine assets. Target customers are primarily early-stage vaccine developers and start-ups in need of specific services and expertise in early-to mid-stage vaccine development.

The particular value proposition of the contract development partnership model consists in the RI functioning as a product development partner for public and private institutions/vaccine developers, including academia, to which an RI and critical high-value services will be provided either free of charge or at a significantly discounted rate.

**Figure 2.**
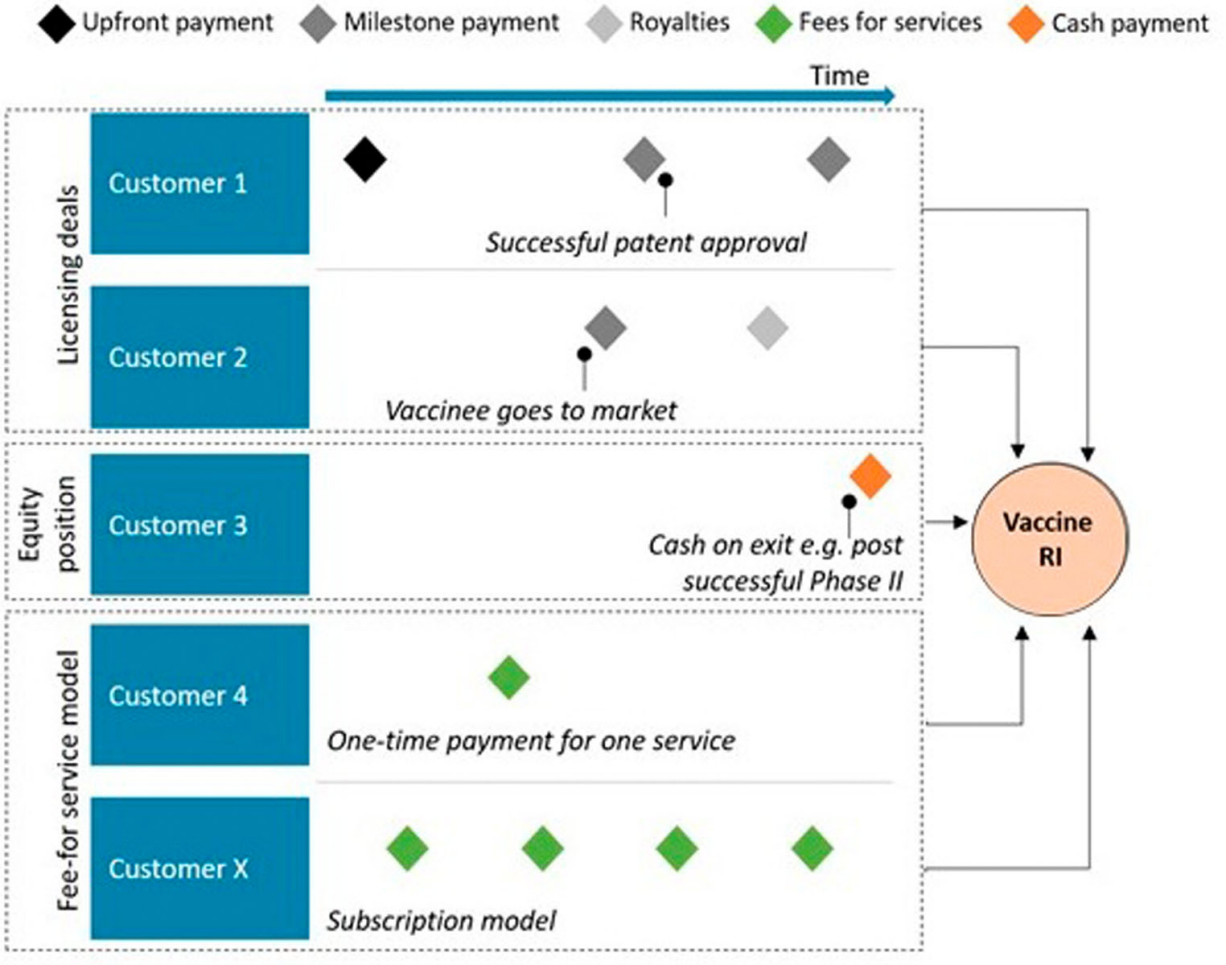
Monetisation model for the contract development partnership.

The monetization model of this business model ( Figure 2) consists in revenues related to the provision of commercial services for which different payment options can be foreseen, such as upfront or milestone payments, royalties, or fees for services, and which will be negotiated on a case-by-case basis.

The most important key steps related to the implementation of the contract development partnership model would mainly involve the definition and establishment of professionalized services/service portfolio, including, for example, the setting up of platforms and operational and managerial procedures, the strengthening of the RI member network to ensure the availability of critical scientific-technical capacities, as well as building project management expertise.

- **Bio-holding incubator model**

**Figure 3.**
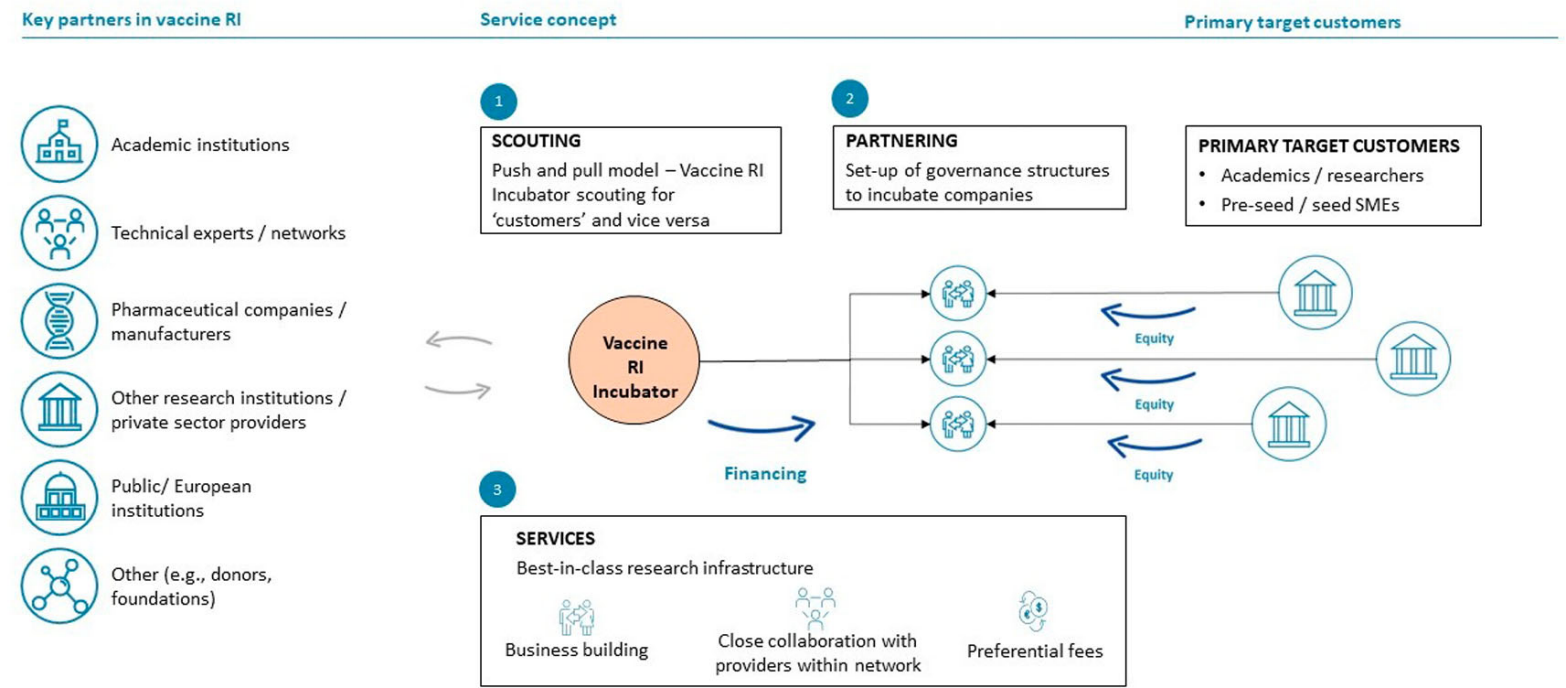
Overview of the functioning of the bio-holding model within the vaccine RI based on the hybrid business model. Blue arrows indicate financial flows, grey arrows scientific-technical or other types of participation and contribution.

Under this model ( Figure 3), the RI will function as an incubator for early-stage vaccine developers by providing funding and active business-building support, including value chain and transversal services. Selected vaccine candidates considered of interest (e.g. due to their indication, market potential, etc) will be in-licensed by the RI. Subsequently, using the financing, scientific-technical expertise and capacities available, the vaccine RI will continue the further development of the in-licensed vaccine candidates down to early- to mid-stage clinical development.

For this model, the RI will be established as a -biotech- holding legal entity that over time will build multiple subsidiaries. The holding company will be constituted by different research organizations (for example, members of the TRANSVAC infrastructure project and other organizations) that thereby will become members or partners in this venture. Each of the subsidiary companies will focus on one individual vaccine candidate (“asset”) that will be in-licensed at late discovery or preclinical development stages and subsequently be further developed in-house, using financing of the bio-holding and the scientific-technical capabilities and expertise of its member organizations. The development of vaccine candidates within the subsidiaries will thus allow them to draw on resources from and to share vaccine development risks with the parent holding company. Target organizations (“target customers”) for the in-licensing of vaccine candidates are small to medium-sized vaccine developers (public and private) with vaccine candidates for further development that require additional external R&D capacities, expertise and financing. The particular value proposition of this model is that the RI will become a business partner for pharma companies and as an RI will conduct R&D to support vaccine assets from discovery/preclinical stages until early-to-mid stage clinical development. Importantly, in addition to scientific-technical support, other value chain services provided by the RI under this model would include the provision of (seed) investments early in vaccine R&D projects, using financial resources from the RI’s own balance sheet.

**Figure 4.**
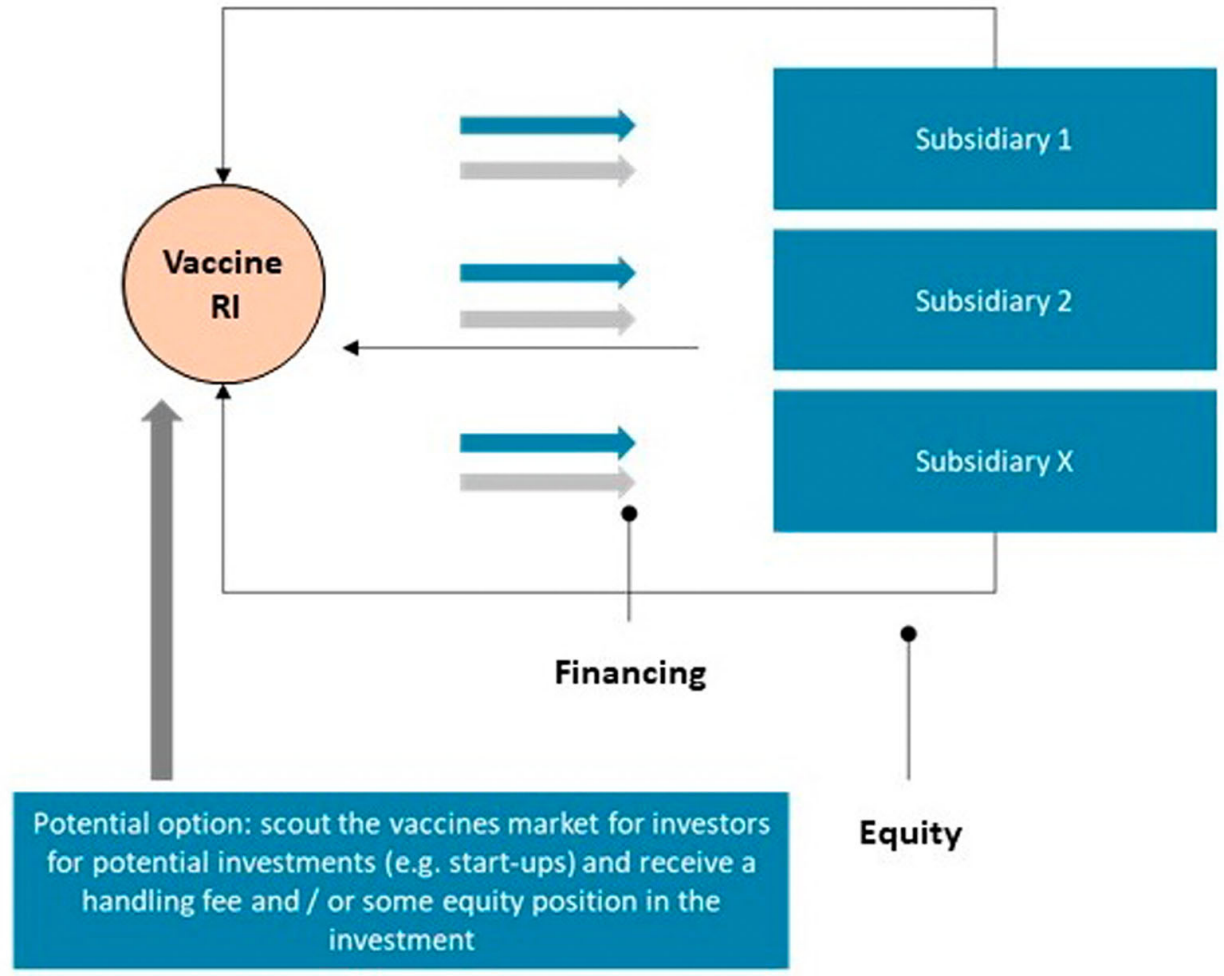
Monetisation model for the bio-holding.

The monetization model under the bio-holding model option is mainly related to the equity deal under which the RI will own a minority stake in the subsidiary companies established which advance the development of the in-licensed vaccine candidates. If and once the vaccine development has successfully completed phase I or phase II clinical testing, the vaccine candidates will be sold to a larger pharmaceutical company such that the equity value growth of the asset will be turned into cash upon exit ( Figure 4). Moreover, the RI will receive a management fee from the investments allocated to it by the external investors to build its portfolio.

The key steps required to implement the bio-holding business model include the need to secure funding/financing (investments) from external investors, such as private equity or venture capital funds, investment banks or others whose financing will be used to cover the costs of the further clinical development of the in-licensed vaccine candidates as well as other operational costs of the RI. An additional requirement will be to build the critical fund management and business building expertise and capabilities within the management team of the RI.

Table 3 provides an integrated summary of the major characteristics of the different model options described above.

**Table 3.** Main features of the two business models integrated in the hybrid model proposed for a sustainable European vaccine RI.

	Hybrid business model	
	Bio-holding	Contract Development Partnership
**Key features**	• Biotech holding company that builds multiple subsidiaries focused on individual assets in order to incubate projects quickly • Drawing on resources from and sharing drug development risks with the parent company	• Provision of scientific-technical services • Option to enter into an equity-based partnership model to co-develop vaccines
**Target customers**	• Small and medium-sized vaccine developers (public and private)	• Small and medium-sized private organizations with need for services in early stages of product development • Academic/public institution vaccine developers partners able to undertake certain types of R&D but in need of specific services/expertise
**Product scope**	• Vaccine candidates in preclinical stages • Mainly human vaccines	• Human and veterinary vaccines
**Value proposition**	• Provide best-in-class RI to conduct R&D • Become business partner for pharma companies	• Providing RI and a pre-defined volume of services (free of charge or at significantly discounted rate to early-stage developments) • Functioning as development partner for public and private institutions (including academia)
**Value chain & service blueprint**	• Seed investments early in R&D projects, utilizing resources from own balance sheet • Support assets from preclinical stages to early-mid stage clinical testing	• Provision of RI and services for transversal activities along the value chain • Potential for collaborative product development including dedicated resources and capacities
**Monetization model**	• Equity-deal with minority ownership position in early-stage vaccine subsidiary companies • Gain through equity growth of the asset until end of life • Optional: Early cash-out to limit long-term risk from competition and price pressure	• Different payment modalities (upfront or milestone payments, royalties, fees-for-services) • Option to free or discounted services in exchange for a small equity stake in the customers’ vaccine assets
**Key steps for implementation**	• Build fundraising and fund management expertise and capabilities • Secure funding (investment) from investors • Build business building expertise and capabilities • Professionalize services	• Build project management expertise • Professionalize services • Build some business coaching expertise and capabilities

### (ii) Socio-economic impact analysis

**Figure 5.**
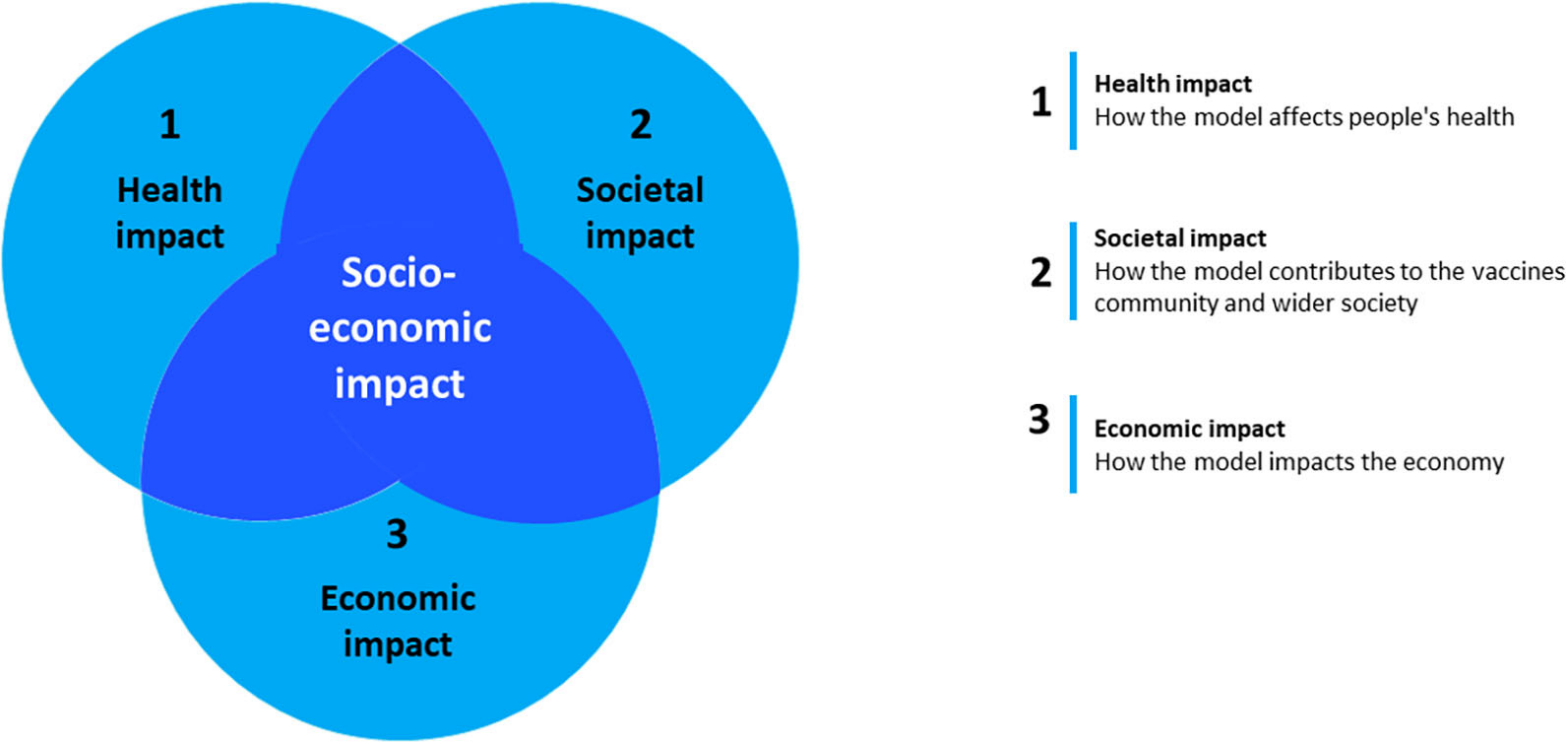
Socio-economic impact of the vaccine RI. The *ex ante* socio-economic impact of the vaccine RI with the proposed hybrid business model was assessed along the three dimensions indicated.

Subsequently, in order to establish a series of explicit, measurable levels of output across a range of activities and to indicate the scale of value creation that is at stake, we undertook an *ex ante* assessment of the socio-economic impact that might be expected from a stable and sustainable European vaccine RI based on the hybrid business model outlined above. The period covered by this assessment includes the first ten years of operations of a vaccine RI based on the business model proposed. The assessment used the KPIs and methodology described in the Methods section in terms of three broad types of dimensions: (i) health impact, (ii) societal impact and (iii) economic impact ( Figure 5).

The health impact dimension refers to innovations in vaccine R&D (such as in terms of tools/technologies/solutions developed, vaccines developed and vaccine-related impacts, e.g. numbers of future deaths prevented and quality of life improvements); the societal impact dimension refers to dissemination activities and new publications, human resources activity, education and training and the value of new vaccines brought to the market; and finally, the economic impact refers to vaccine industry, expected numbers of new jobs created, business and related outputs, impact on SMEs, impact on research institutes and networking activities. Apart from these dimensions, the KPIs were split across three categories in order to indicate the time horizon of their measurement, selected to best mirror the progress of the RI, namely (i) short-term, (ii) long-term cumulative impacts, and (iii) an operational metrics to be used for monitoring of the RI execution. The estimated impacts for all three dimensions are summarized in Table 4.

**Table 4.** Impacts estimated for sustainable European vaccine RI based on the business model proposed. Impacts across three different dimensions have been estimated for the first 10 years of the RI’s operation period, in addition to other operational impacts for the tracking of activities. Each measure indicated in the left column corresponds to an individual KPI.

Measure of potential impact (KPI)	Impact estimate
**Estimated health impact**	
Expected number of new vaccines in clinical development	Total of 15 new vaccines supported across preclinical down to phase 2 stages (via bio-holding; during first ten-year period), leading to one novel vaccine entering market by the end of the ten-year period (after de-risking and accounting for development duration)
Expected number of future deaths and severe cases prevented	80,000 to 1.1 million deaths averted per single vaccine (low and high impact scenario, respectively; with more vaccine candidates in pipeline beyond ten-year timeframe allowing to scale that impact further)
Expected DALY improvement	Between +2.5 million and +10 million DALYs saved per single vaccine (low and high impact scenario, respectively; with more vaccine candidates in pipeline beyond ten-year timeframe allowing to scale that impact further)
**Estimated societal impact**	
Number of new jobs created (within subsidiary companies)	100–150 new positions
Percentage of researchers who have been through training who report an improvement in their knowledge base/knowledge capital created	75% reporting ‘good’ and 50% reporting ‘excellent’ based in post-course surveys
Media appearances (Television, radio, press and online)	30 separate appearances a year
Number of research partners trained through vaccine infrastructure	135 partners trained during ten-year period
**Estimated economic impact**	
Expected revenue generated	€180 million at the end of first ten-year period
Expected value of funding attracted, including services/grants	€102 million during first ten-year period
Expected value of licensing deals	€159 million during first ten-year period
Expected cash inflows at bio-holding and venture level	€53 million during first ten-year period
Number of SMEs created (this is also a proxy for number of licensing deals)	15 SMEs during ten-year period
Number of scientific services provided	Total of 33 projects supported via contract development partnership model during first ten-year period with 50 instances of services provided
Number of new patents issued	One patent issued every four years
**Estimated operational impact**	
Expected number of new publications	Five new publications a year from the third year of operation onwards
Expected number of new scientific services established as an offering to researchers	One new service offered every two years (a total of five over the ten-year period)
Number of vaccine projects supported	A total 15 vaccine projects supported during first ten-year period
Organisation of stakeholder and investor meetings	12 meetings per year

(a) **Health impact**

The ultimate objective of this initiative is to support and enhance the vaccine R&D ecosystem in order to address both the financial ‘translational valley of death’ (the funding gap between early research and clinical trials that makes the translation of basic science into clinical candidates so challenging) and a more general capabilities gap in early development stages. This will be realized over a longer period of time, and as a result is expected to produce more novel vaccine candidates that successfully advance through clinical trials and into medical practice.

The expected number of new vaccines in development will reflect candidates whose origination and development have been supported by the vaccine infrastructure activities. This requires tracking vaccines through each stage of their development – in other words, the number of candidates that have reached key successive milestones (i.e. the number of new vaccines proceeding to first-in-human trials, reaching proof-of-concept stage and entering the market). According to an analysis of annual trends data ^5^, the average length of the development cycle from phase 1 to market is ~7.5 years (outside of pandemic and seasonal situations). This has therefore to be a long-term metric and one which encompasses the entirety of the first ten years of the initial RI operation. Our expectation of one novel in-market project being enabled in ten years is consistent with the vaccine infrastructure producing one late-stage novel project over its first five years of operation. This is also consistent with 15 new early-stage vaccines being supported throughout this time period. Considering the average length of vaccine development (for complex projects it can take 12 years or more from preclinical to end of phase 3), the majority of these projects will not have reached clinical adoption by the end of that first decade ( Terry *et al.*, 2018). For projects that would progress to the mid-late stage clinical testing of vaccines, industry-average success rates are applied to account for inevitable attrition to arrive at approximately one novel infrastructure-supported vaccine reaching approval within the first decade.

Such a vaccine would likely be followed by other candidates, so impact could scale up beyond that first decade as more projects progress through clinical trials. If we consider the range of vaccine-specific probabilities of success and likely development cycle time, between 2 and 4 of the 15 supported assets would be expected to reach successful launch. This impact could be further expanded by improvements to existing licensed vaccines (e.g., other routes of administration or to make production more cost-effective). Such incremental changes typically do not require a full development process and hence such new products can be adopted more quickly and at a lower risk. Several such products could thus reasonably be anticipated over the following 10-20 years (even though the prospective health impact of vaccine improvement is more variable and harder to estimate given that it would depend on the needs left unmet by current vaccines).

The true impact of novel vaccines reaching market is their actual benefit for patients and health systems. This can be expressed as the expected number of future deaths, severe cases prevented and the expected disability-adjusted life year (DALY) improvement. For one new vaccine estimated to be introduced to market after the first ten years (accounting for the aforementioned time and risk), the actual impact on population health may be estimated. The accuracy of such an estimate is subject to significant uncertainty given variations in the prevalence, virulence, mutation rates and severity of the kinds of disease that might be targeted by such a vaccine – each of which affect burden, mortality, achievable vaccine effectiveness and adoption rate.

Several approaches exist for modelling the impact achieved by recently-introduced vaccines. Based on models published in the medical literature, we suggest two scenarios may be helpful to consider in arriving at an understanding of the scale of health impact achievable for a single new vaccine: a COVID-19-based case to illustrate the potential benefits of a novel vaccine in a pandemic setting, and an influenza vaccine efficacy improvement scenario to illustrate the benefits achievable for common endemic diseases (even ones already treated with vaccines that are not yet fully optimized).

The real-world impact of a novel product might fall somewhere between these low and high outcomes, whereas potentially being further enhanced by more of the 15 novel vaccine candidates supported by this effort reaching market over a longer time period. Other, smaller incremental projects might be supported that in turn might reach market sooner (and with lower risk), but in turn their potential health impact could be expected to be more incremental -typically only improving upon existing vaccines- and as a result too elusive to model separately.

***High-impact scenario (pandemic vaccine)***

Based on recent assessments made with respect to COVID-19, the introduction of vaccines prevented approximately 4.3 million deaths (from 8 December 2020 to 8 December 2021) in the European region alone ( Watson *et al.*, 2022) ^6^. Acknowledging that within the assessment timeframe there were in total four novel SARS-CoV-2 vaccines approved for use across the European region, we estimate that on average, the annual impact of a single vaccine in this case was over a million lives saved in Europe.

In parallel and considering the overall effect of COVID-19 in terms of DALYs, a recent estimate concluded the DALY impact of COVID-19 in just Scotland and only in 2020 was 102,350 ( Wyper *et al.*, 2022). This calculation reflects the specific demographic and healthcare characteristics of just one region of the UK but considering the size of the population of Scotland in relation to that of the entire WHO European region (approximately 0.6% of the total), an equivalent effect for the region might be very approximately estimated as being more than 150 times as great, or over 15 million DALYs.

The DALY decrease linked to vaccination can be anticipated to be in proportion to the ratio of deaths prevented to total projected deaths as modelled ( Watson *et al.*, 2022) (72% in first year of vaccination accounting for vaccination coverage and efficacy). Applying this to our approximate estimate of 15 million for the DALY effect of a new pathogen resembling SARS-CoV-2 implies a vaccine impact in excess of 10 million DALYs in the region, and if four vaccines were again to be introduced and with approximately equal effect, an impact for a single vaccine on the order of 2.5 million DALYs ( Table 4). The conversion of deaths to DALYs may vary for different infections, for example SARS-CoV-2 resulted in large numbers of individuals with Long COVID.

***Low-impact scenario (influenza vaccine efficacy improvement)***

The second scenario assumes an efficacy improvement and administration optimization for an existing (but not optimized) vaccine against a common but-usually-less severe endemic disease. It is based on modelling influenza and its seasonal vaccine of 2017-18 (characterized by lower than usual efficacy) compared to a more optimized variant ( Sah *et al.*, 2018). Compared to no vaccination, a low-efficacy vaccine already drives significant health benefit -for instance, a 20% efficacy flu vaccine administered at ~40% coverage is projected to avert more than 21 million infections, ~130,000 hospitalizations, 61,812 deaths, and 2.2 million DALYs in the US ( Sah *et al.*, 2018). With efficacy increased to 40%, the scale of health benefit can be doubled. Scaled for Europe (based on the total population of Europe and the USA as of 2020), this would imply approximately 28 million infections, 175 thousand hospitalizations, more than 80,000 deaths, and 3 million additional life years saved.

The health benefits represented by DALYs and deaths averted are further strengthened by the economic impact on the health systems. This is evidenced by the cost-effectiveness of vaccination programs, with most costing less than $50 per life gained and thus orders of magnitude cheaper than the prevention of many non-infectious diseases such as diabetes or hypertension ( Bloom, 2011; Ehreth, 2003) ( Table 4).

(b) **Societal impact**

The vaccine infrastructure can deliver positive societal impact both directly and indirectly. The indirect societal consequences of a successful vaccine deployed at scale are potentially enormous. This set of indirect impacts has not been assessed.

In terms of direct impact, it is possible to anticipate the number of new jobs created. By attributing new jobs solely to those in new SMEs as a consequence of a more assured pathway between innovation and clinical trials, and assuming an average of 5-10 employees for each, this would imply 100-150 new positions over ten years. The broader perception of the European vaccine development industry can be enhanced by a vigorous publication record and effective communications (see below). Further, a target can be set for the percentage of researchers undergoing training who experience an improvement in their knowledge base/knowledge capital, as quantified by post-course surveys. These targets are set as 75% of trainees reporting at least a ‘good’ level of improvement in their knowledge base in post-course surveys, and 50% of trainees reporting ‘excellent’ improvement, based on historical feedback from previous training courses organized in the context of the TRANSVAC project ^7^ (unpublished data).

More broadly, a target may be reasonably set for media appearances at 30 separate appearances a year, based on past TRANSVAC dissemination rates (this including press releases, website launches and social media campaigns; and also a target for the number of research partners trained through the vaccine infrastructure (assuming nine training courses in total with an average of 15 attendees per course, this can be set at 135 over the ten-year period in question). All these activities help ensuring a positive momentum for the sector by building awareness, disseminating findings and contributing to reputation building for the sector ( Table 4). This is particularly critical for the introduction of new vaccines whose benefits could be undermined by false information on social media, supported by the increase in vaccine hesitancy in some communities.

(c) **Economic impact**

Any vaccine that is successful in preventing or ameliorating serious disease is likely to have a significant and sometimes very substantial indirect economic impact (see, for example, Atkins *et al.*, 2018; Sandmann *et al.*, 2022; Portnoy *et al.*, 2023). Positive effects are also going to be observed for the local life science innovation ecosystem and industry – those effects being achieved even before any successful products reach the market and contribute to increased competitiveness and economic health. For the purposes of this socio-economic assessment analysis, however, only direct economic impact is considered.

The KPIs selected for measuring economic impact would likely require observation and targeting in respect to expected revenue generated. Our business plan modelling suggests total revenue on successful exits after ten years amounting to €159 million, but given the long-term nature of vaccine development, these revenues would be most likely to accrue towards the end of the period. To put this value in context, average annual European sales of novel non-COVID vaccine products were €175 million in 2021 ^8^. The expected value of funding attracted is assumed to be €100 million over ten years. The expected value of licensing deals is aggregated with an expected revenue generation of €159 million over ten years (i.e. much or all of the revenues obtained from the initiative will be realized in the form of licensing deals).

These are not formal targets but rather reasonable estimates of what could be expected based on the extrapolation into the future of the activities conducted under the TRANSVAC project. Revenue and investment values contribute directly to the reinvigoration of local R&D ecosystems. Moreover, it is assumed the vaccine infrastructure’s activity may also lead directly to SME creation by facilitating access to funding and capabilities – with the target for the number of SMEs created being 15 over the first ten years of the initiative.

In addition to large deals and the birth of new SMEs, the vaccine infrastructure can also provide a range of scientific services. These may vary significantly in scope from technical capabilities and execution of experiments to more comprehensive offerings including study design and a more complex execution capability. The precise services offered will be finalized in the future with consideration for the demand for specific services during the past TRANSVAC projects as well as stakeholder surveys we conducted. The most popular services have included antigen expression/production, adjuvant formulation testing, animal models for infectious diseases, immunocorrelates analysis, and regulatory support.

Within the ten-year period, a contract development partnership model could be expected to support a total of 33 projects, with 50 instances of services provided. The predicted revenue stream generated by these services is small, estimated at ~€0.2 million per year (in line with currently observed inflow from ongoing projects), contributing an additional €2 million to the overall consortium revenue over ten years. The main economic value, however, is in the invigoration of the ecosystem and enabling academic and industry innovators to access capabilities they usually would be unable to maintain in-house (and that might otherwise be inaccessible, considering the limited scope of contract research organisations (CROs) services offered within early-stage vaccine R&D).

In addition to financial metrics, the vaccine infrastructure can also make a direct contribution to the generation of new innovations and intellectual property by supporting vaccine R&D. As part of this, the number of new patents issued would be expected to equal TRANSVAC’s historic performance (i.e., one approximately every four years). Such patents represent meaningful improvements in R&D processes (e.g., formulation or technical methods) and thus facilitate development of novel products across the ecosystem ( Table 4).

(d) **Operational impact**

The most important consequences of the vaccine infrastructure are only fully measurable over the long term due to the characteristics and length of the vaccine development process, which must account for manufacturing, rigorous clinical trials and regulatory evaluations. It is therefore especially important to identify operational activity variables which can be reported on a more frequent basis, but which can be reasonably associated with long-term success. The expected number of publications may be observed and targeted (five a year on a two-year time lag to allow for the process of paper preparation, submission, review and acceptance based on TRANSVAC’s historic experience) and the expected number of new scientific services established as an offering to researchers (one new service offered every two years, coming to a total of five over the ten-year period). The number of vaccine projects supported at any one time can be tracked (five to ten projects, as mentioned above). Finally, a robust level of engagement can be targeted in terms of the organization of stakeholder and investor meetings (12 a year, to cover a variety of combinations of stakeholders and/or investors) ( Table 4).

## Discussion

RIs are fundamental enablers of science, research, innovation and education. They facilitate cutting-edge scientific investigations and support the development of innovative solutions to various challenges. However, RI are also known to be notoriously expensive in their establishment and maintenance, and the costs for their establishment alone often range from millions to even billions of Euros, depending on the scientific discipline and type of infrastructure. Very frequently, public funders bear most of this burden. This is also true for most if not all already of the existing RI in Europe. The major source of funding for the existing RI that are part of the ESFRI Roadmap, is public funding provided by different European Union (EU) Member States’ governments although most of them through their operations aim to generate revenues e.g. by providing commercial services. Most of these RI also obtain additional funding via competitive EU and other types of grants (i.e., additional public funding). So, although probably all of the existing RI have revenue comprising a mixture of financing from different sources, the largest share by far of their total income comes from public funding sources without which none of the RI would be able to survive or operate.

Being acutely aware of the levels of financial commitments and other responsibilities that this near-absolute dependance of RIs on public money entails, governmental funders and decision makers have a keen interest in promoting the development of alternative business models that may allow RIs to become sustainable and operate more independently of public funding.

Striving for sustainability has also been the major rationale underlying the TRANSVAC design study. A well-designed and stable vaccine R&D RI could play a major role in tackling existing challenges and in advancing innovation in the field. We have demonstrated this in the successful implementation of the TRANSVAC projects, that—thus far with the exclusive financial support of the EU—obtained important achievements. For example, the TRANSVAC and TRANSVAC2 projects have together reviewed 158 applications for free services and approved 88 projects from 19 countries. Nearly a quarter (18) of those projects involved services provided by multiple institutions. TRANSVAC2 has also organised two rounds of a new, free 14-course vaccinology training program. More than 400 trainees from academia and industry attended, helping to fill an important training gap in the field ( Geels *et al.*, 2015; Martin *et al.*, 2023). Beyond the provision of scientific-technical services, TRANSVAC was also charged with conducting a feasibility study with the aim of developing and proposing an alternative business model for a sustainable European vaccine RI. As the first step in this exercise, we undertook a detailed gaps-and-needs analysis of vaccine R&D in Europe ( Jungbluth *et al.*, 2022), the outcomes of which informed the subsequent steps. The most important findings from this assessment that shaped the development of alternative business models for a vaccine RI were the lack of funding and financing available for vaccine R&D, the need for expertise and guidance to vaccine developers, and the difficulty in accessing critical technologies, platforms and other key infrastructure.

These findings present an opportunity for a new initiative and formed the basis for the hybrid business model we have presented here. This hybrid model offers a mixture of scientific services for potential vaccine products and support for business development. Regarding vaccine development, the vaccine RI we envision will offer a portfolio of services emphasizing high-value scientific-technical services, provided on a commercial basis with different payment modalities or the option to enter into an equity-based partnership model. Importantly, beyond the provision of services to “external” vaccines, the bio-holding model contained in our business model will go further and in-license into the vaccine RI selected vaccine candidates that have been identified and considered of interest. Financing will be raised from external investors such as private equity or venture capital funds, and investment banks to fund in-house development of these in-licensed vaccine candidates through phase I and phase II clinical testing. If these clinical steps are successful, the vaccine assets will be sold to pharma companies for late-stage development and commercialization, with the equity share corresponding to the vaccine RI in these assets being the major revenue stream for the infrastructure in the long term.

Importantly, by combining two complementary business models, our hybrid model offers the advantage of two different types of revenues. One is based on income linked to the provision of scientific-technical services on a commercial basis; income for the RI in this case will mainly consist of a brokerage fee charged for the mediation of services between the customer and the service provider. The second, and more important revenue in the long term, is linked to the expected equity growth of the in-licensed vaccines (of which the RI would hold a minority stake) that will generate cash upon sale to pharma companies. Although smaller in amount compared to the income from the bio-holding model, the revenue via the contract development partnership model has the advantage of offering a continuous stream of income which can cover the operating costs of the RI (e.g. human resource-related costs), especially in the first years of the RI. As mentioned above, the bio-holding model income is expected to be more important in the long run, but is only expected at a later stage of the implementation of the vaccine RI due to the more complex requirements, the risk of failure of any single vaccine candidate, and the time required to reach clinical trials. The contract development partnership model thus provides a smoother entry for the establishment of the bio-holding model and represents an overall measure of financial risk reduction. Moreover, the different types of requirements linked to the establishment of the two different models will enable a relatively fast and straightforward establishment of the contract development partnership in the early stages of the overall venture, subsequently allowing the RI to concentrate more efforts on the establishment of the bio-holding model. The bio-holding model clearly is the more demanding venture to establish due to, for example, the need to mobilize significant external financing and to scout and manage the in-licensing of vaccine candidates. For a vaccine RI based on our proposed business model to function in the long run, it is important to emphasize that it will only work if the bio-holding model can be implemented successfully, as the revenue stream based on the contract development partnership only would not be sufficient to sustain the operation and running of the RI.

As mentioned before, the development of this business model was informed by stakeholder consultations we conducted to ensure that the model aligned as closely as possible to stakeholder needs and interests. For the same reason, once this model had been developed and refined, we returned to several key stakeholders, including researchers from public and private organizations, investors and investment banks, to pitch the model to them. The feedback we received from this exercise was largely positive and encouraging. Overall, the model we have developed was considered relevant and feasible—but challenging—to implement.

The *ex ante* impact assessment we conducted shows that a vaccine RI based on the model we have developed is likely to have a very important impact along the three dimensions we explored (the health, societal and economic spheres). These dimensions are discussed individually below. However, it must be acknowledged that vaccine development is inherently uncertain and despite utilizing the best available evidence and making the estimates in good faith, these estimations remain subject to significant potential positive or negative variance. Although there is a lot of literature and thinking around ways of measuring the impact (including *ex ante*) of aRIs (see, for example, ESFRI, 2023), we are not aware of any *ex ante* socio-economic impact assessment for a biomedical RI to the level of detail and depth we have undertaken for a vaccine RI.

In the health dimension, our estimates indicate that - using our investment target of €100 million - a total of 15 new vaccines can be supported from preclinical stages to phase 2 clinical testing, of which during the 10-year time frame of our estimations we expect 1 vaccine candidate can be sold to pharma industry (for the assumptions underlying our estimations, such as vaccine development timelines, attrition rates, costs, etc., see the Methods section). For estimating the eventual public health impact of the vaccines supported by the vaccine RI, we have used two different scenarios, one based on a high-impact case (such as a pandemic vaccine similar to COVID-19 vaccines), and another scenario based on a low-impact case (such as a seasonal influenza vaccine with improved efficacy). The expected numbers of future deaths and severe cases prevented for the low and high impact scenarios, respectively, range from 80,000 to 1.1 million deaths averted per single vaccine. This estimation does not include any of the other vaccine candidates in the vaccine RI pipeline beyond the 10-year timescale of our estimations; if those were also considered then expected impact would be higher. Regarding the improvement of DALYs, we expect between +2.5 million and +10 million DALYs saved per single vaccine for the low- and high- impact scenarios, respectively (also here considering only the 10-year timeframe).

One has to keep in mind that these estimated health impacts only relate to the vaccine candidates that will be in-licensed and developed in-house by the vaccine RI (via the bio-holding model) and do not include any “external” vaccines whose development will be supported by the commercial scientific-technical services provided by the RI (via the contract development partnership model). Although many of these “external” vaccines can be expected to have additional positive impacts on public health in the long term, estimating this impact proved too speculative and therefore was not included in our overall impact assessment. Overall, considering the level of initial investment our venture would require, we consider that our model for a vaccine RI has the potential to achieve a very substantial positive impact on public health.

Regarding the societal impact, we estimate that between 100-150 new positions will be created in direct relationship to the model we propose. On one hand, there will be a certain number of new positions linked to the management headquarter of the RI itself. Most of the new positions, however, will be linked to the establishment of the subsidiary companies that will be established under the parent holding company. As we foresee that a single company will be established for each vaccine candidate in-licensed to the vaccine RI, for the 10-year period of the currently planning we estimate a total of up to 15 subsidiaries. Each of these subsidiaries will be relatively small in size but require a certain number of scientific-technical staff. The number of subsidiary companies could also be somewhat lower, as some vaccine candidates developed in-house by the vaccine RI may not require formal in-licensing and establishment of a new, dedicated subsidiary company.

The other impacts in the societal dimension relate to training offered via the vaccine RI and the overall number of media appearances of the venture. These estimations are directly extrapolated from data from the TRANSVAC projects and are considered highly reliable. Overall, we consider the realistic societal impact as defined here to be significant, although less critical than the health impact due to the nature and size of the proposed venture.

For the economic dimension, we have estimated the impacts linked to both parts of our business model (the bio-holding and contract development partnership aspects). Based on our experience providing scientific-technical services through the TRANSVAC projects and the experience of already existing RI in the biomedical field that provide commercial services, the direct economic impact related to the provision of services by a vaccine RI can be estimated in a very reliable manner. This relates both to the number of commercial services provided and the corresponding revenue. As mentioned above, as the vaccine RI we are proposing for the service provision functions mainly as a broker, and the direct income of the central infrastructure related to this business model essentially corresponds to a brokerage fee. Although representing only a minor part of the overall revenue generated by the RI in the long term, it will nevertheless be important for starting the entire venture and facilitating the transition towards the establishment of the bio-holding model and also addresses an important existing need in the vaccine R&D community. However, most of the revenue is eventually expected to result from the bio-holding model, namely the growth in equity that has been generated between in-licensing and selling the vaccines after their further development. This business model is based on large pharma companies buying these vaccine assets after successful phase I or phase II clinical stages. Vaccines must have commercial potential to maintain relevance for large pharma. Consequently, at least during the first ten years or so of its existence, a vaccine RI based on our model for the bio-holding venture will--and must--mainly target vaccines of relevance for high income markets in order to become sustainable. Once sufficient profit has been generated by the vaccine RI, eventually the capital available can also be used to in-license and further develop vaccines for neglected diseases with more limited commercial perspectives. Vaccines of this kind could also be supported earlier in case financing from philanthropic investors or public funding are mobilized for the vaccine RI to allow direct support for products with less market potential.

## Conclusions

Our consortium has attempted to develop a business model for a sustainable vaccine RI that would be able to operate without a dependence on public funding and estimated its impact using the most reliable data possible and based on widely accepted assumptions and existing evidence. Nevertheless, substantial variations and uncertainties are inherent to this type of modeling and *ex ante* assessment. Still, both our model and the estimations will become increasingly better and reliable the more we advance with this venture and with the particular steps we will achieve. Uncertainties will be replaced by facts, and new data will become available both from our own work, as well as from those of others working in this field.

To conclude, we believe that our model can be viable and that the expected impacts are sufficiently high to merit advancing. The next step in the establishment of a vaccine RI at this particular moment is the selection both of the country for the RI’s headquarters, and -linked to this- the selection of a legal entity option considered suitable for our particular model. We are currently in the process of defining the key criteria and aspects to be used for assessing and comparing different legal entity options, considering aspects such as the role and liability of members/shareholders, costs of establishment, reporting, audit and accounting requirements, supervision by the authorities, and taxation issues. Given that the mission of the envisioned vaccine RI is to accelerate vaccine development in the interest of public health and societal benefits, legal entity options will be prioritized and explored that allow the establishment of the RI as a non-profit venture.

Vaccine development is directly linked to a better future for Europe and beyond. We believe that a well-designed and sustainable European vaccine RI has the potential to promote the development of the industry and a significant regional and global health impact.

## Data Availability

No data are associated with this article.

## Footnotes

^1^

www.transvac.org

^2^
Pharmaprojects. https://pharmaintelligence.informa.com/products-and-services/clinical-planning/pharmaprojects

^3^
Evaluate. www.evaluate.com/

^4^
Pharma Deals: https://www.pharmadeals.net/about

^5^
Evaluate. www.evaluate.com/

^6^
WHO European Region comprises: Albania, Andorra, Armenia, Austria, Azerbaijan, Belarus, Belgium, Bosnia and Herzegovina, Bulgaria, Croatia, Cyprus, Czech Republic, Denmark, Estonia, Finland, France, Georgia, Germany, Greece, Hungary, Iceland, Ireland, Israel, Italy, Kazakhstan, Kyrgyzstan, Latvia, Lithuania, Luxembourg, Malta, Moldova, Monaco, Montenegro, Netherlands, North Macedonia, Norway, Poland, Portugal, Romania, Russia, San Marino, Serbia, Slovakia, Slovenia, Spain, Sweden, Switzerland, Tajikistan, Turkey, Turkmenistan, Ukraine, United Kingdom and Uzbekistan.

^7^

www.transvac.org

^8^
Evaluate. www.evaluate.com/
