# Supplementary material for: Potential business model for a European vaccine R&D infrastructure and its estimated socio-economic impact
Source: F1000Res. 2023 Oct 24;12:1401. [Version 1] doi: 10.12688/f1000research.141399.1 (PMC10828550; doi:10.12688/f1000research.141399.1)
Supplement: Peer review history

# Reviewer report: Reviewer response for version 1

**DOI:** 10.5256/f1000research.154839.r234705

**Published:** 2024-02-15

**Authors:** Massimo Florio

**Affiliations:** 1 Universita degli Studi di Milano, Milan, Lombardy, Italy

I have read this article with interest because of my own work for the European Parliament and other bodies about the need of European Biomedical Research Infrastructure, and more recently about the evaluation of the EU response to Covid 19 pandemic. 

 I provide some links: 

 a)https://www.europarl.europa.eu/cmsdata/250660/Study%20presentation%20STOA%20panel%2016.12.2021.pdf

 b) htts://www.europarl.europa.eu/thinktank/en/document/IPOL_STU(2023)740072

 I would also mention this new study by Gamba et al: 

 c) https://www.europarl.europa.eu/stoa/en/home/news/details/new-stoa-study-on-better-access-to-medic/20200309CDT03501.

 The Authors may take advantage of the literature, empirical results, and policy options discussed there. Moreover, they may consider that on July 2023 the European Parliament has voted a recommendation to the EC and the Council.

 d) "605.  Calls on the Commission and the Member States to create a large-scale, mission-oriented, public European health R&D infrastructure which operates in the public interest to manufacture medicinal products of health and strategic importance for healthcare, in the absence of existing industrial production, in order to support the EU to overcome market failure, guarantee security of supply and prevent possible shortages of medicines, while contributing to greater preparedness for facing new health threats and emergencies;" https://www.europarl.europa.eu/doceo/document/TA-9-2023-0282_EN.html 

 Finally, they are by sure aware of the R&D  implications of the EUPharma Strategy and the ongoing revision of the legislation on medicines. In the next months the European Parliament needs to vote on it position for future negotiations with the EC and the Council.

 In this context, the proposals by the Authors, stemming from their experience in Transvac, a Horizon2020 funded consortium, are welcome. However, their ambitions are limited and the hybrid model they propose is perhaps not entirely appropriate to the objective of a EU wide RI.

 It seems that on one side the model would be a service provider to third parties, such as pharma stat-ups, SMEs, Universities and so on. The Authors list some services but it is not entirely clear to me why the proposed RI with, as it seems, limited own staff and budget, should make a difference in the European Panorama with existing Contract Organizations, except probably because the services would be sold at low cost, reflected in minimal revenues in the sketched business plan. I understand this venture as a not-for-profit entity, perhaps a distributed RI, but  there are others in the EU, for example for clinical trials, etc. Thus the Authors should show why they would be able to offer something not already available and precisely what and to whom. 

 Having said this the Authors turn to an entirely different model, where the RI would act as sort of venture capitalist for certain vaccine projects, mainly in the earliest clinical studies. They forecast that in ten yers a vaccine would emerge from this approach, targeted to something in the scale of  between the SARS COV-2 vaccines or an incremental influenza  virus. This is very vague indeed, and the spectrum is so wide that one cannot understand the appropriateness of the strategy. 

 Should the Authors want to revise this paper or write a follow up my suggestions are as follows:

 a) Firstly, take advantage of the previously mentioned studies and frame their proposal in the context of ongoing policy debate in the EU about pharma R&D

 b) Secondly, consider that  if they are proposing a public- private but not for profit initiative they also need to show why it should be competitive with exisiting organisations in terms of services offered: they may for example consider a benchmark organisation and discuss their assessment 

 c) Finally, for the hub model, the Authors seem to basically adopt a business model that may overlap with the activity of venture capitalists on one side, or even the activity of some public bodies, such as HERA and the EIB, which are also proposing to provide support and seed capital etc in certain R&D investment projects. 

 d) The issue of Intellectual Property Rights arising from the hub activities should be also more discussed.

 I understand that what I am suggesting goes probably beyond the Authors' intentions, but as mentioned they can try and be more ambitious. They certainly have first hand evidence about R&D needs, lack of certain services and of capital, and about the overall panorama to better place their proposals in the context.

 To be clear, I have nothing against an hybrid model, but as it stands there are two subsequent models in one paper rather than a synergic combination of activities. Perhaps the Authors may deal with this issue by starting from the beginning with a more complete proposal.

Is the work clearly and accurately presented and does it cite the current literature?

Partly

If applicable, is the statistical analysis and its interpretation appropriate?

Partly

Are all the source data underlying the results available to ensure full reproducibility?

No

Is the study design appropriate and is the work technically sound?

Partly

Are the conclusions drawn adequately supported by the results?

No

Are sufficient details of methods and analysis provided to allow replication by others?

No

Reviewer Expertise:

Public economics

I confirm that I have read this submission and believe that I have an appropriate level of expertise to confirm that it is of an acceptable scientific standard, however I have significant reservations, as outlined above.

# Reviewer report: Reviewer response for version 1

**DOI:** 10.5256/f1000research.154839.r234689

**Published:** 2024-01-30

**Authors:** Sana Zakaria

**Affiliations:** 1 RAND Europe, Cambridge, England, UK

1. Spelling correction for poliomielitis required. It should be spelled poliomyelitis.

 2. It is unclear what the scope of the proposed European vaccine R&D infrastructure would be. The intro mentions that RIs range in scope so it would be helpful to define what scope has been considered in this assessment. I.e. is it inclusive of translational research and preclinical/clinical testing? This is somewhat alluded to at the start of the results but should be more explicit as these boundaries could heavily impact the monetisation models presented.

 3. The monetisation models are really interesting but there is little assessment of whether these models would be financially viable and how the upfront RI is expected to be financed within the hybrid access model. It would be good to have some worked up examples on cost of vaccine research and establishment of RI against the benefits anticipated. In terms of upfront establishment of the RI, the models proposed here do not talk about this but rather develop models that could provide varied revenue streams for sustaining/maintaining existing RI. This should be specified upfront as the following sentence is misleading: "The model we have developed integrates two different types of business models that would provide the infrastructure with two different types of revenue streams which would facilitate its establishment and would be a measure of risk reduction."

 4. The financial modelling details would be valuable to include as a supplementary appendix to understand the basis of the estimations proposed. 

 5. I really like the nuancing of health impacts as presented especially in different settings.

Is the work clearly and accurately presented and does it cite the current literature?

Yes

If applicable, is the statistical analysis and its interpretation appropriate?

Not applicable

Are all the source data underlying the results available to ensure full reproducibility?

No

Is the study design appropriate and is the work technically sound?

Partly

Are the conclusions drawn adequately supported by the results?

Partly

Are sufficient details of methods and analysis provided to allow replication by others?

Partly

Reviewer Expertise:

health and technology policy research, experimental medicine research

I confirm that I have read this submission and believe that I have an appropriate level of expertise to confirm that it is of an acceptable scientific standard, however I have significant reservations, as outlined above.

# Reviewer report: Reviewer response for version 1

**DOI:** 10.5256/f1000research.154839.r234698

**Published:** 2024-01-30

**Authors:** Ingrid Kromann

**Affiliations:** 1 Coalition for Epidemic Preparedness Innovations (CEPI), Oslo, Norway

I have carefully reviewed the manuscript and I appreciate the opportunity to provide constructive feedback on the paper.

 Summary: The paper addresses an important topic—coordinated vaccine development infrastructure—and highlights the significant need for sustainability in this area. The manuscript effectively discusses the challenges faced during the initial stages of vaccine development, focusing on the transition from preclinical research to clinical research, commonly known as the first valley of death. However, to support the business model the paper could be strengthened to provide a more comprehensive understanding of late stage development, matchmaking with industry and commercialization, particularly regarding the vaccine portfolio and region in scope (second valley of death).

 Specific Suggestions:

**1:** The paper would benefit from a more detailed explanation of the specific regions and vaccine areas the proposed infrastructure is intended to address. It is crucial to clearly outline if it is the unmet needs and/or EPI vaccines the infrastructure aims to target. This clarification will enhance the paper's relevance and applicability to diverse stakeholders involved in vaccine and business model development.

**2:** While the manuscript extensively covers the challenges associated with the first valley of death, it briefly touches upon the critical second valley of death, which involves the transition from late-stage development to commercialization. The paper should delve deeper into this aspect, providing a more thorough analysis of the business model's dependency on licensed/commercial products. A comprehensive discussion on the funding requirements and the importance of strategic matchmaking for successful transition is crucial. Additionally, detailing the industry's interest in vaccines and emphasizing the correlation between vaccine area selection and business model success will strengthen the manuscript.

**3** The manuscript briefly mentions the industry's interest being dependent on the demand for commercial products. Expanding on this point and elucidating how the selection of vaccine areas influences industry engagement will provide a more nuanced perspective. The paper should discuss how aligning vaccine development with market demand enhances the likelihood of attracting funding and industry support.

**4** The conclusion could be strengthened by summarizing the key initiatives needed and emphasizing the interdependence of geographical focus, thematic relevance, and business model considerations in the proposed vaccine development infrastructure. It is essential to reiterate the paper's contributions and provide a clear call to action for policymakers, researchers, and industry stakeholders.

 In conclusion, while the manuscript addresses a crucial topic and provides valuable insights into the challenges of vaccine development infrastructure, further refinement in the areas mentioned above will enhance the paper's clarity, depth, and overall impact.

Is the work clearly and accurately presented and does it cite the current literature?

Yes

If applicable, is the statistical analysis and its interpretation appropriate?

Not applicable

Are all the source data underlying the results available to ensure full reproducibility?

Partly

Is the study design appropriate and is the work technically sound?

Yes

Are the conclusions drawn adequately supported by the results?

Partly

Are sufficient details of methods and analysis provided to allow replication by others?

Yes

Reviewer Expertise:

Vaccine CMC/manufacturing development

I confirm that I have read this submission and believe that I have an appropriate level of expertise to confirm that it is of an acceptable scientific standard, however I have significant reservations, as outlined above.

# Reviewer report: Reviewer response for version 1

**DOI:** 10.5256/f1000research.154839.r218383

**Published:** 2023-11-08

**Authors:** Wolfgang Fecke

**Affiliations:** 1 European Science Foundation, Strasbourg, Grand Est, France

This is a very interesting study for the development of a business plan for a vaccine research infrastructure which could become relevant also for other biomedical not-for-profit organizations. Some aspects are less well covered, in particular the area of competing with commercial CROs in the vaccine field. How many customers would find it more attractive to work with an academic not-for-profit rather than with a professional service provider remains unclear.

 The KPIs listed in Table 2 rely mostly on unpublished data and are therefore not easy to use by others (hence my 'partly' answer in terms of data reproducibility) although some numbers are mentioned in Table 4. In that respect, the number of new patents appear fairly low (one every four years), given the number of projects (33) and incubated companies (15) within 10 years.

 One minor point is a certain redundancy of information in Figures 1-4, these could be streamlined.

 n the discussion the costs of running the infrastructure are mentioned in the sense that the service model cannot cover these costs. It would be helpful to have actual figures available on which this statement is based. What is the estimated income from the brokerage fees, and what are the personnel and other operational costs of the infrastructure? For instance, costs for general administrative or financial services through third parties could be obtained. Additional revenue stream of the infrastructure are possibly overheads derived from EC-funded projects - these are not taken into account at all although historical figures from the TRANSVAC projects exist.

 Finally, the funding gap at least in the first years of the infrastructure could still be covered by membership contributions from countries although it is laudable that the authors attempt to design a fully sustainable model without public funding. Maybe it would be worth mentioning in the discussion that this option exists.

 Overall, the article will be useful also for other biomedical infrastructures to support their business plans - as the authors mentioned in their discussion, another publicly available study which such detailed numbers does not exist for an infrastructure or would be at least hard to find.     

Is the work clearly and accurately presented and does it cite the current literature?

Yes

If applicable, is the statistical analysis and its interpretation appropriate?

Not applicable

Are all the source data underlying the results available to ensure full reproducibility?

Partly

Is the study design appropriate and is the work technically sound?

Yes

Are the conclusions drawn adequately supported by the results?

Yes

Are sufficient details of methods and analysis provided to allow replication by others?

Yes

Reviewer Expertise:

Research infrastructures, chemical biology, drug discovery

I confirm that I have read this submission and believe that I have an appropriate level of expertise to confirm that it is of an acceptable scientific standard.
